# Relevance of minor discrepancies at second pathology review in gynaecological cancer

**DOI:** 10.3332/ecancer.2019.929

**Published:** 2019-05-13

**Authors:** Lucas Minig, José Manuel Bosch, Carmen Illueca, Cristina Zorrero, José Miguel Cárdenas-Rebollo, Julia Cruz, Ignacio Romero

**Affiliations:** 1 Gynecology Department, CEU Cardenal Herrera University, 46115 Valencia, Spain; 2 Gynecology Department, Instituto Valenciano de Oncología (IVO), 46009 Valencia, Spain; 3 Pathology Department, Instituto Valenciano de Oncología (IVO), 46009 Valencia, Spain; 4 Department of Applied Mathematics and Statistics, CEU San Pablo University, 28003 Madrid, Spain; 5 Medical Oncology Department, Instituto Valenciano de Oncología (IVO), 46009 Valencia, Spain; a https://orcid.org/0000-0001-7648-5604

**Keywords:** second opinion, pathology, diagnosis, gynaecological cancer, referral cancer centres, quality of care

## Abstract

**Aim:**

To determine the incidence of discrepancy rate between the initial pathology diagnosis and referral diagnosis in women with gynaecological cancer.

**Methods:**

A retrospective observational study was performed including all consecutive patients with gynaecological cancer referred and who underwent pathologic review between January 2013 and May 2017. Discrepancies were minor when future treatment was not altered or major when the treatment was modified.

**Results:**

A total of 259 patients were included. The original diagnosis was ovarian cancer (*n* = 126, 48.6%), endometrial cancer (*n* = 84, 32.4%), cervical cancer (*n* = 43, 16.6%) and vulvar cancer (*n* = 6, 2.3%). Eighteen women (6.9%) had major discrepancies and 69 patients (26.6%) had minor discrepancies. The main reason for the minor discrepancy was tumour grade or histology subtype. Regarding ovarian cancer, 13 out of 16 patients had minor discrepancies at histology subtype among serous, endometrioid, mucinous or undifferentiated tumours. The main issue for the minor discrepancy in patients with cervical cancer was among different subtype of cervical adenocarcinoma. Minor discrepancies due to tumour grade were also observed in 14, 19, 8 and 3 patients with endometrial, ovarian, cervical and vulvar cancer, respectively.

**Conclusions:**

A second pathology review also adds valid information in those cases with minor discrepancies leading to a difference in patients´ counselling regarding follow-up and prognosis.

## Introduction

Discrepancy in the pathology diagnosis is possible among different pathologists [1]. In fact, the disagreement seems to be more frequent for gynaecological pathologies [2]. Thus, secondary pathology review for gynaecological cancer is a common practice at referral centres [3]. It is defined as the review of pathology specimens, by a second pathologist, which is usually initiated at the request of the treating clinician, multidisciplinary tumour board, quality control protocol or as standard practice to review all cases at a specialised centre prior to treatment [4]. According to the American Society of Clinical Pathology, pathology review is recommended as an important component of total quality programmes in diagnostic surgical pathology, being a key aspect in the assurance of patient safety for tissue-based diagnoses [5].

Some authors have found that discrepancies between the initial diagnosis of gynaecological cancer and the second opinion leading to modification of the treatment, referred to as a major discrepancy, can range between 0.6% to 13.5% of cases [1, 3, 6–11]. However, there is little information in the literature regarding discrepancies that do not modify future treatments but can alter the prognosis of the disease, the patient´s counselling, as well as the follow-up. Therefore, this study aims to determine the incidence of discrepancy rate between the initial and referral pathology diagnosis in women with gynaecological cancer.

## Materials and methods

### Study design

A retrospective observational study was performed in all consecutive patients with gynaecological cancer referred to the Instituto Valenciano de Oncología (IVO), in Valencia, Spain. Patients who attended for a second opinion and who underwent pathologic review between January 2013 and May 2017 were included. Patients with non-described information at the initial pathology diagnosis, such as tumour grade or histology subtype, were also included. Patients with insufficient sample to establish a second pathology review were excluded from this study.

### Data collection

Information regarding the original pathology diagnosis was abstracted from the original pathology report and included the type of sample (biopsy or tumour specimen), site of tumour, histology subtype and grade of differentiation. The second pathology diagnosis was collected from the patient´s chart and included: site of tumour, histology subtype, the grade of tumour differentiation, as well as the modification on the patient´s treatment. The type of discrepancy was classified in accordance with other authors [1, 3, 7, 9] as: minor discrepancy, when it did not impact on future clinical practice (including those patients previously treated or with missing information at the original pathology report); and major discrepancy, when it did modify the patient´s future planned treatment.

### Pathology examination at IVO

One pathologist specialising (C.I.) in gynaecological cancer performed the pathology review. In case of any type of discrepancy, a second pathologist (J.C.) reviewed the diagnosis. Both pathologists have more than 20 years of experience in gynaecological cancer. The diagnosis was based on criteria described in textbooks, as well as the WHO Classification of Tumours of Female Reproductive Organs [12, 13]. Additional immunohistochemical stains were performed following institutional protocols to achieve the final pathology diagnosis or as part of specific clinical trials. The information was presented in a weekly multidisciplinary tumour board to establish the appropriate treatment plan.

### Literature review

A literature search in PubMed was performed using the following criteria: ((‘pathology’[Subheading] OR ‘pathology’[All Fields] OR ‘pathology’ [MeSH Terms]) AND slide [All Fields] AND (‘review’ [Publication Type] OR ‘review literature as topic’ [MeSH Terms] OR ‘review’ [All Fields])) AND ((‘gynaecology’ [MeSH Terms] OR ‘gynaecology’ [All Fields] OR ‘gynaecological’ [All Fields]) AND (‘neoplasms’ [MeSH Terms] OR ‘neoplasms’[All Fields] OR ‘cancer’[All Fields])) AND (‘01/May/1997’[Date - Publication]: ‘30/November/2017’[Date - Publication]). The searches were limited to ‘English’ and ‘human’. Additional literature was searched through cross-references of the retrieved articles to extract all relevant literature available.

The inclusion criteria were: (A) Cases series or cohort studies; (B) Studies must describe minor and major discrepancy as described above between initial and second opinion diagnosis. All abstracts of the studies obtained were evaluated for eligibility by two of the authors (Lucas Minig and José Manuel Bosch).

### Statistical analysis

Frequencies and proportions were used as summary statistics for categorical variables. Time interval to the second opinion was compared by using the chi-square test, where *p* < 0.05 was considered as significant differences. Statistical analysis was performed using the IBM SPSS version 20.0 programme.

## Results

Pathology review was required in 259 patients with gynaecological cancer who attended the IVO for a second opinion, representing the analysed patients in this study. Almost half of the patients had ovarian cancer (*n* = 126, 48.6%), while 84 patients (32.4%) had endometrial cancer, 43 (16.6%) had cervical cancer and six women (2.3%) had vulvar cancer. The type of sample analysed included: biopsy in 105 patients (40.5%), dilatation and curettage (all endometrial cancer) in ten cases (3.8%), uterus in 40 women (15.4%), ovary (all ovarian cancer) in 103 patients (39.7%) and cervical cone (all cervical cancer) in one case (0.4%). Immunohistochemical analysis was required in 182 women (71%). Discrepancy among the initial and referral diagnosis was observed in 87 patients (33.5%); of them, 69 women (26.6%) had minor discrepancy and 18 patients (6.9%) had a major discrepancy. Median (range) time interval to obtain the second opinion was 4 (0–38) days, without significant differences among patients with major and minor discrepancies (Table 1).

Table 2 describes the characteristics of patients with minor and major discrepancies in different types of gynaecological cancer. Among patients with endometrial cancer, minor and major discrepancies were diagnosed in 16 (19%) and seven patients (8.3%), respectively. A total of 14 women (87.5%) had minor discrepancies at tumour grade, one case (6.2%) at histology subtype and one at both. One patient (14.2%) had major discrepancies in tumour grade, while the other six cases (85.7%) were found in histology and tumour grade. Regarding patients with ovarian cancer, 19 out of 35 patients (54.2%) with minor discrepancies had tumour grade disagreement, nine women had histology discrepancy, while the remaining seven cases had differences at both histology characteristics.

### Minor discrepancy by histology

A total of 23 patients had minor discrepancies in histopathology and they are detailed in Table 3. Among the 16 patients with the ovarian disease, the discrepancy between serous, endometrioid and mucinous histology was noted in nine cases.

### Minor discrepancy by grade

Nineteen patients with ovarian cancer had minor discrepancy by tumour grade only. In 16 cases, the tumour was graded at IVO since this information was not described at the original pathology report; two patients were downgraded from serous high-grade (G2) to serous low-grade (G1) epithelial ovarian cancer (EOC); while one patient was upgraded from low-grade (G1) to high-grade (G3) EOC.

Minor discrepancies regarding tumour grade in women with endometrial cancer were observed in 14 patients. Tumour grade information was not described in nine pathology reports; while three patients with G1 endometrioid endometrial cancer were upgraded to G2 tumours. The last two cases had G3 and G2 tumours, and they were downgraded to G2 and G1, respectively.

Regarding cervical cancer, the tumour was graded at IVO in two out of the eight patients with minor discrepancy by tumour grade. Three patients were downgraded from G3 to G2 or G1, while three cases were upgraded. Finally, tumour grade was established at IVO in two patients with vulvar cancer; while the tumour was upgraded from G1 to G2 in one additional patient with vulvar cancer, all affected by squamous cell carcinoma.

### Major discrepancy

The characteristics of 18 patients with major discrepancies are described in Table 4. Of note, five patients had uterine cancer where the discrepancy was modified from endometrioid to serous or clear cell endometrial cancer. (cases # 8, 9, 10, 11, 12) Discrepancy at the origin of the tumour was diagnosed in two cases. One patient had an initial mucinous EOC and was modified at IVO to metastatic gastric cancer (Krukenberg`s tumour). The second case, initially diagnosed as urothelial/endometrial carcinoma at a uterine cervical biopsy, was modified to a poorly differentiated (G3) squamous cervical cancer by using additional immunohistochemistry such as: CK20, CK7, WT-1, P53, P16 and P63.

### Literature review

The literature review identified eight retrospective studies, which evaluated the level of discrepancy between the initial pathology diagnosis and the referral diagnosis after slides review. The studies were published between 1998 and 2014 and included a total of 3,463 patients with gynaecological cancer. Six studies analysed all types of gynaecological cancer, while two additional studies evaluated ovarian and vulvar cancer separately. Table 5 shows the minor discrepancy in women with endometrial cancer ranged between 6.3% and 24%, while major ones between 0.6% and 13.5%. The range of minor discrepancies in ovarian cancer was 3.7%–38% and the major discrepancy was 5%–9.2%. Women with cervical cancer had a range of minor and major discrepancies of 2.5%–36% and 0.7%–8.3%, respectively.

## Discussion

This study showed a discrepancy in the pathology diagnosis between the original centre and IVO in 33.5% of patients with gynaecological cancer. Of them, 6.9% had major discrepancies and 26.6% had minor discrepancies that did not changed the patients’ treatment. The distribution of the latter group was 19%, 27.7%, 9.3% and 50% of patients with endometrial, ovarian, cervical and vulvar cancer, respectively.

Observing major discrepancies between the initial and the referral pathology diagnosis in women with gynaecological cancer is considered the most important goal. Other authors have observed a major discrepancy rate ranging from 0.6% to 13%, which is in range with the present study (Table 5) [1, 3, 6–11]. The definition of ‘major discrepancy’ varies in each study. While the great majority of those studies defined major discrepancy as a disagreement that altered the treatment [1, 3, 7, 9, 10], one study also included cases in which the prognosis was altered as well [6]. This could be one of the explanations as to why the range found is broad among the different studies previously published.

To our knowledge, this is the first study that specifically describes the pathology diagnosis discrepancy that does not alter the treatment in patients with gynaecological cancer. However, this type of disagreement, usually classified as a minor discrepancy, [1, 3, 7, 9] can also give relevant information, leading to modifications on the patient´s counselling in terms of prognosis of the disease, type of follow-up, while reducing patient/family anxiety. Minor discrepancies in gynaecological cancer can include a group of pathology characteristics such as: tumour grade, histology subtype, depth of stromal invasion, as well as tumour origin. It is also important to highlight that some patients asked for a second opinion after completing the initial treatment. Thus, in some cases in the present study (patients # 2, 3, 4, 5, 6, 13, 16, 17 of Table 4), even though a second pathology review found a major discrepancy that would have modified the initial treatment strategy, patients did not receive any treatment modification because they asked for a second opinion after receiving full treatment.

Modification in tumour grade differentiation can alter clinical management and counselling in different types of gynaecological cancers. Thus, while this information rarely modifies the management of cervical cancer, tumour grade can be important in endometrial and ovarian malignancies. Our study identified two women initially diagnosed with high-grade serous ovarian cancer, which was then downgraded to low-grade EOC. Even though this was classified as a minor discrepancy, these patients were counselled differently regarding their prognosis and the potential chemoresistance of their disease [14].

The present study also noted the minor discrepancy in the histology subtype in 23 out of 259 patients (Table 3). Given that many of these patients already arrived after the initial treatment or at relapse of the disease, the treatment was not modified. However, the patients´ counselling regarding future follow-up and prognosis was significantly changed. Histology subtype is a common area of discrepancy in gynaecological cancer [3, 10]. As in our study, other authors have found a discrepancy in serous, endometrioid or sarcoma histology in women with uterine cancers [3]. In addition, disagreement in histology subtype was also noted in other studies in ovarian cancer [10]. A sub-analysis of a prospectively randomised phase III study analysed 454 patients with an original diagnosis of epithelial ovarian carcinoma. All original slides were requested, and a specialised central pathology review was performed by experienced pathologists in gynaecological cancer. A total of 15 (3.3%) patients were misdiagnosed as having invasive cancers, while histology discrepancy among EOC was noted in 128 (28.2%) patients [10].

The current study is limited, however, by the fact that only selected slides and/or paraffin blocks were sent for revision at IVO. One interesting study compared 468 cases with selected slides with 128 cases in which all slides were available. Even though all medical conditions were included, the authors noted less disagreement in the ‘selected slide group’ than in the ‘all slide group’ (12% versus 18%, *p* = 0.03) [15]. While it seems that sending all slides is better, selecting the appropriate slides to be sent is time-consuming for the original pathologists and often requires a second opinion at the original institution in order to ensure that the original diagnosis is correct. One proposed alternative is to write in the original pathology report which slides are most appropriate to confirm the diagnosis. Another matter of concern is whether some reliable slides should be retained or not at the original institution for legal purposes for a potential lawsuit in the future. This situation might be previously arranged between both institutions to return the material once the second opinion has been made at the referral institution [15].

In either setting, once a discrepancy is noted, it seems appropriate to perform a second opinion at the same referral institution [16]. In case of persistence, contacting the original pathologist to discuss the point of disagreement and to confirm the discrepancy or not is recommended [17]. This situation certainly will improve the overall quality of pathologists´ performance by sharing knowledge and information and will be finally translated into a better quality of care for patients.

Another area of concern, according to histology and grading modification, would be that, although at this very moment only the use of poly ADP ribose polymerase (PARP) inhibitors is driven by both conditions, current efforts are being carried out to select emerging targeted therapies based on histology and grade, not only on tumour location.

Even though the present study has strengths, which include the first study in its nature specifically designed to describe pathology discrepancies that do not lead to modifications in the treatment in women with gynaecological cancer, our results have limitations. First, the retrospective collection of data might be associated with pathologists’ misinterpretation and missed information, as well as with lost patients. Second, even though the patients’ characteristics are homogeneous, the number of analysed cases might be small to reach strong results and conclusions; therefore, it should be interpreted with caution. Third, a subset of patients might not be referred to the second institution for further management or second opinion.

## Conclusion

In conclusion, the incidence of minor and major discrepancies in women with gynaecological cancer was 26.6% and 6.9%, respectively. The second pathology review can not only modify the treatment strategy in some cases but can also give relevant information regarding different disease prognosis, follow-up and improve patient´s counselling. Therefore, a second pathology review performed by expert pathologists at referral centres should be performed in gynaecological cancer patients who ask for a second opinion.

## Conflict of interest

All the authors declare that they have no competing interests.

## Ethical approval

The Institutional Review Board approved the execution of the study.

## Informed consent

All patients signed informed consent to analyse the pathology samples. Specific informed consent was not required in this study.

## Figures and Tables

**Table 1.** Patients’ characteristics.

Characteristics	*N* = 259
Type of cancer	
Ovarian	126 (48.6%)
Endometrial	84 (32.4%)
Cervical	43 (16.6%)
Vulvar	6 (2.3%)
Type of sample analysed	
Biopsy	105 (40.5%)^1^
D&C (all endometrial cancer)	10 (3.8%)
Uterus	40 (15.4%)
Ovary (all ovarian cancer)	103 (39.7%)
Cervical cone (cervical cancer)	1 (0.4%)
Pathology characteristics	
IHQ requirement	182 (71%)
Discrepancy	87 (33.5%)
Minor	69 (26.6%)
Major	18 (6.9%)
Time interval to the second opinion, in days	Median (range)^2^
All patients (*n* = 259)	4 (0–22)
Minor discrepancy (*n* = 82)	4 (0–22)
Major discrepancy (*n* = 10)	4.5 (0–14)

**Legends**: D&C: dilatation and curettage; IHQ: Immunohistochemistry requirement

1 includes 23 cases of ovarian cancer

2 *p*-value: 0.276

**Table 2.** Minor and major discrepancies among different types of gynaecological cancer.

	Endometrial cancer (*n* = 84)		Ovarian cancer (*n* = 126)		Cervical cancer (*n* = 43)		Vulvar cancer (*n* = 6)	
	Minor 16 (19%)	Major 7 (8.3%)	Minor 35(27.7%)	Major 7 (5.5%)	Minor 15 (34.8%)	Major 4 (9.3%)	Minor 3 (50%)	Major 0 (0%)
Discrepancy								
Grade	14 (87.5%)^a^	1 (14.2%)	19 (54.2%)^b^	0	8 (53.3%)^c^	0	3 (100%)	0
Histology	1 (6.2%)	0	9 (25.7%)	6 (85.7%)	4 (26.6%)	1 (25%)	0	0
Both	1 (6.2%)	6 (85.7%)	7 (20%)	1 (14.2%)	1 (6.6%)	2 (50%)	0	0
Origin	0	0	0	1 (14.2%)	2 (13.3%)	1 (25%)	0	0
IHQ	15 (93.7%)	5 (71.4%)	32 (91.4%)	2 (28.4%)	6 (40%)	2 (50%)	0	0
Sample analysed								
Biopsy	7 (43.7%)	4 (57.1%)	6 (17.1%)	-	14 (93.4%)	4 (100%)	3 (100%)	0
D&C	2 (12.5%)	1 (14.2%)	-	-	-	-	-	-
Uterus	7 (43.7%)	2 (28.5%)	-	-	1 (6.6%)	0	-	-
Ovary	-	-	29 (82.8%)	7 (100%)	-	-	-	-

**Legends**: IHQ: Immunohistochemistry requirement; D&C: dilatation and curettage

References:

(a) Nine patients due to missing information at the original pathology report.

(b) Sixteen patients due to missing information at the original pathology report.

(c) Six patients due to missing information at the original pathology report

**Table 3.** Minor discrepancy by histology.

	Initial histology	Grade	Final histology	Grade
Ovarian cancer				
1	Squamous	*	Clear cell	*
2	Serous borderline	N-Ap	Mucinous borderline	N-Ap
3	Serous EOC	*	Endometrioid EOC	*
4	Serous EOC	*	Endometrioid EOC	*
5	Serous EOC	NA	Mucinous EOC	2
6	Serous EOC	NA	Undifferentiated EOC	3
7	Endometrioid EOC	NA	Serous EOC	H-G
8	Endometrioid EOC	*	Serous EOC	*
9	Endometrioid EOC	*	Serous EOC	*
10	Endometrioid EOC	NA	Serous EOC	H-G
11	Endometrioid EOC	*	Serous EOC	*
12	Endometrioid EOC	*	Undifferentiated EOC	*
13	Undifferentiated EOC	*	Serous EOC	*
14	Undifferentiated EOC	*	Serous EOC	*
15	Neuroendocrine, hypercalcaemic small cell	*	Undifferentiated EOC	*
16	Neuroendocrine, Large cell	*	Serous EOC	*
Endometrial cancer				
17	Endometrioid	*	Mucinous	*
18	Leiomyosarcoma	3	Undifferentiated sarcoma	3
Cervical cancer				
19	Endometrioid	*	Adenosquamous	*
20	ADC serous	*	ADC mucinous	*
21	ADC	1	ADC endometrioid	2
22	Adenosquamous	*	Squamous	*
23	ADC villoglandular	*	ADC endometrioid	*

**Legends:** EOC: epithelial ovarian cancer; NA: not available; N-Ap: not applicable; H-G: High-grade. ADC: Adenocarcinoma

* : grade concordance

**Table 4.** Characteristics of patients with major discrepancies.

	Specimen type		Original diagnosis	Second opinion	IHQ	Treatment alteration
Ovarian cancer						
1	Ovary	Mucinous, EOC		Krukenberg (gastric)	Yes	Surgery cancelled
2	Ovary	Serous borderline		High-grade Serous EOC	Yes	Surgery modified
3	Ovary	Serous borderline		Endosalpingiosis	No	Surgery modified
4	Ovary	Serous EOC		Benign cystadenoma	No	Surgery modified
5	Ovary	Mucinous EOC, G1		Serous cystadenoma	No	Surgery modified
6	Ovary	Immature teratoma		Mature teratoma	No	Surgery modified
7	Ovary	Serous, EOC, G3		Yolk sac tumour	Yes	Different CT
Endometrial cancer						
8	Uterus	Endometrioid, G1		High-grade serous carcinoma	Yes	Surgical re-staging
9	D&C	Endometrioid intraepithelial		High-grade serous carcinoma	Yes	Surgery modified
10	Biopsy	Endometrioid, G1		Clear cell	Yes	Surgery modified
11	Biopsy	Endometrioid intraepithelial		High-grade serous carcinoma	Yes	Surgery modified
12	Biopsy	Endometrioid, G1		High-grade serous carcinoma	Yes	Surgery modified
13	Biopsy	Serous, grade not described		Endometrioid, G2	No	Surgery-CT modified
14	Uterus	Undifferentiated sarcoma		Low-grade endometrial stromal sarcoma	Yes	Radiotherapy cancelled
Cervical cancer						
15	Biopsy	Adenocarcinoma		Neuroendocrine carcinoma	Yes	CT added
16	Biopsy	Squamous, G2		High-grade intraepithelial lesion	No	Surgery modified
17	Biopsy	Squamous, G2		High-grade intraepithelial lesion	No	Surgery modified
18	Biopsy	Urothelial/endometrial carcinoma		Squamous, G3	Yes	Different chemotherapy

**Legends:** EOC: epithelial ovarian cancer; CT: Chemotherapy; D&C: dilatation and curettage; IHQ: Immunohistochemical requirement

**Table 5.** Studies reporting minor and major discrepancies in women with gynaecological cancer.

		Endometrial cancer		Ovarian cancer		Cervical cancer		Vulvar cancer	
Author, year	N	Minor	Major	Minor	Major	Minor	Major	Minor	Major
Santoso *et al.* [1]	720	18/170 (10%)	1/170 (0.6%)	10/105 (9.5%)	5/105 (5%)	45/289 (15.5%)	2/289 (0.7%)	18/156* (11.5%)	5/156* (3.2%)
Chan *et al*. [7]	569	34/242 (14%)	20/242 (8.2%)	9/54 (16.6%)	5/54 (9.2%)	27/233 (11.6%)	12/233 (5.1%)	2/35 (5.7%)	2/35 (5.7%)
Selman *et al.* [6]	295	6/94 (6.3%)	7/94 (7.4%)	1/34 (2.9%)	3/34 (8.8%)	3/119 (2.5%)	1/119 (0.8%)	0/43	3/43 (7%)
Chafe *et al*. [8]	599	60/296 (20%)	40/296 (13.5%)	22/122 (18%)	10/122 (8.2%)	46/127 (36%)	8/127 (6.3%)	9/33 (27.2%)	2/33 (6%)
Khalifa *et al.* [3]	351	38/159 (24%)	18/159 (11.3%)	13/55 (23.6%)	4/55 (7.2%)	11/84 (13%)	7/84 (8.3%)	3/28 (10.7%)	0/28
Eskander*et al.* [9]	279	11/100 (11%)	7/100 (7%)	2/53 (3.7%)	3/53 (5.6%)	5/86 (5.8%)	5/86 (5.8%)	6/31 (19.3%)	3/31 (9.6%)
Kommoss *et al.* [10]	454	-	-	128/454 (28%)	31/454 (7%)	-	-	-	-
Beugeling*et al.* [11]	121	-	-	-	-	-	-	0/121	2/121 (1.6%)
Current series 2018	259	16/84 (19%)	7/84 (8.3%)	35/126 (27.7%)	7/126 (5.5%)	15/43 (34.8%)	4/43 (9.3%)	3/6 (50%)	0/6 (0%)

* Including 43 vaginal cancer
